# Sphingosine-1-Phosphate and Its Receptors: A Mutual Link between Blood Coagulation and Inflammation

**DOI:** 10.1155/2015/831059

**Published:** 2015-10-29

**Authors:** Shailaja Mahajan-Thakur, Andreas Böhm, Gabriele Jedlitschky, Karsten Schrör, Bernhard H. Rauch

**Affiliations:** ^1^Institut für Pharmakologie, Universitätsmedizin Greifswald, Felix-Hausdorf Strasse 3, 17487 Greifswald, Germany; ^2^Institut für Pharmakologie und Klinische Pharmakologie, Universitätsklinikum Düsseldorf, Universitätsstrasse 1, 40225 Düsseldorf, Germany

## Abstract

Sphingosine-1-phosphate (S1P) is a versatile lipid signaling molecule and key regulator in vascular inflammation. S1P is secreted by platelets, monocytes, and vascular endothelial and smooth muscle cells. It binds specifically to a family of G-protein-coupled receptors, S1P receptors 1 to 5, resulting in downstream signaling and numerous cellular effects. S1P modulates cell proliferation and migration, and mediates proinflammatory responses and apoptosis. In the vascular barrier, S1P regulates permeability and endothelial reactions and recruitment of monocytes and may modulate atherosclerosis. Only recently has S1P emerged as a critical mediator which directly links the coagulation factor system to vascular inflammation. The multifunctional proteases thrombin and FXa regulate local S1P availability and interact with S1P signaling at multiple levels in various vascular cell types. Differential expression patterns and intracellular signaling pathways of each receptor enable S1P to exert its widespread functions. Although a vast amount of information is available about the functions of S1P and its receptors in the regulation of physiological and pathophysiological conditions, S1P-mediated mechanisms in the vasculature remain to be elucidated. This review summarizes recent findings regarding the role of S1P and its receptors in vascular wall and blood cells, which link the coagulation system to inflammatory responses in the vasculature.

## 1. Introduction

Sphingosine-1-phosphate (S1P), a highly active lipid mediator, exhibits a broad range of cellular activities including proliferation, survival, adhesion, and migration [1, 2]. S1P is critical for mammalian cardiac development and for maturation of the systemic circulatory system [3]. These biological actions are carried out by predominantly intracellularly produced S1P via sphingosine kinase (SphK), of which two isoforms SphK1 and SphK2 exist [4, 5]. Moreover, S1P has emerged as an intracellular second messenger involved in regulation of cell proliferation and in mobilization of internal calcium stores by a protein kinase C independent pathway [6]. Further reports suggest that S1P found within the extracellular space is not merely derived from intracellular generation but biosynthetic enzymes of the S1P metabolism appear to subsist in the extracellular space [7]. Indeed, the majority of studies have focused on the functions of extracellular S1P. This “outside the cell” S1P acts in an autocrine or paracrine manner as an agonist for a unique family of G-protein-coupled receptors which to date comprises the five S1P receptors (S1PRs) S1PR1–S1PR5 [8, 9]. Extracellular S1P regulates proliferation and migration of vascular endothelial cells (ECs) [10] and smooth muscle cells (VSMCs) [11] and critically determines lymphocyte egress and angiogenesis [12]. Both S1P and S1PRs regulate vascular tone either by directly modulating the smooth muscle layer or by stimulating ECs to release bioactive molecules which regulate VSMCs responses in a paracrine manner [13].

The levels of S1P in plasma and tissues are tightly regulated by the balance between its synthesis by sphingosine kinases and degradation [2, 14]. The role of S1P and the processes involved in its biosynthesis, that is, regulation of the metabolizing enzymes, for controlling vascular integrity has been studied thoroughly* in vitro* and* in vivo* [15]. Vascular proliferative disorders such as atherosclerosis and persistent proinflammatory challenges of the vessel wall [16] are characterized by the activation of the coagulation cascade and platelet activation, both processes which elevate local S1P concentrations [17]. This may play an important role in directing immune cells to sites of local injury and directly links the coagulation system to S1P-mediated inflammatory responses* in vivo*. After vascular injury, the coagulation cascade is initiated by activating the clotting factors X (FXa) and ultimately thrombin, which are both key regulators of subsequent tissue repair and remodeling [18, 19]. FXa-mediated thrombin generation initiates and is itself amplified by subsequent platelet activation, finally leading to cleavage of fibrinogen and eventually the formation of the mural thrombus [20, 21]. In addition to their physiological function in hemostasis, the clotting proteases thrombin and FXa are also accountable for clinically relevant pathological responses such as postphlebitic inflammatory and tissue repair reactions [16, 22]. The biological effects of FXa and thrombin are mediated via a family of G-protein-coupled receptors, protease-activated receptors 1, 2, 3, and 4 (PAR-1–PAR-4) [23, 24]. Thrombin initiates signaling through PAR-1, PAR-3, and PAR-4, while FXa acts via PAR-1 and PAR-2. Previous reports, including studies from our group, have reported that PARs stimulate VSMCs proliferation and migration, modify the composition of the extracellular matrix of blood vessels, and mediate proinflammatory responses in the vessel wall [25–28]. Because proliferation and migration of VSMCs are considered key events in the development of atherosclerosis and vascular remodeling, these cellular effects of thrombin and FXa may directly contribute to the pathogenesis of vascular diseases such as progression of atherosclerosis and restenosis after vascular injury. In addition, recent studies highlight numerous interactions between blood coagulation and the S1P signaling system [17, 29].

This review discusses the recent findings concerning the role of S1P and its receptors in vascular and blood cells which are interlinked with the coagulation system. Particularly, hemostasis-related mechanisms which increase local S1P availability and the regulation of PAR receptor expression by S1P are highlighted. Elucidating the complex interactions between blood coagulation and the S1P signaling network further may bear the potential to discover and develop novel targets for the therapy of inflammation-prone vascular diseases.

## 2. Biosynthesis, Degradation, and Functions of S1P in the Vascular System

S1P biosynthesis is tightly interlinked with the metabolism of ceramide. Ceramide is formed either* de novo* from serine, palmitoyl CoA, and fatty acid or from breakdown of membrane-resident sphingomyelin [30, 31]. Ceramide is further converted to sphingosine by enzymatic action of ceramidase. Finally, the bioactive lysophospholipid S1P is produced by phosphorylation of sphingosine. This reaction is catalyzed by the two sphingosine kinase isoenzymes SphK1 and SphK2. Maintaining a balance between S1P generation and degradation is critical for regulation of cell growth and plays a key role in pathological processes such as carcinogenesis [32]. S1P degradation is achieved via reversible dephosphorylation by two S1P-specific phosphatases (SPP1 and SPP2) or irreversible hydrolysis by S1P lyase. S1P exerts actions either by binding to its intracellular targets or through its specific receptor in autocrine, paracrine, and/or endocrine manner [31].

**Figure 1:**
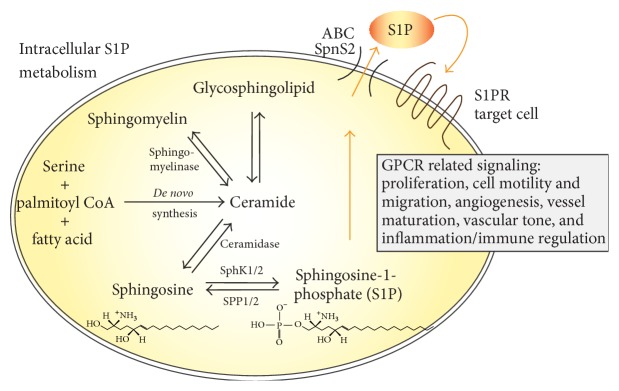
Synthesis of S1P and functions in the vascular system.* De novo* ceramide synthesis in general originates from condensation of serine, palmitoyl CoA, and fatty acid, a multistep enzyme catalysed process. Ceramide can be converted reversibly into sphingomyelin by sphingomyelinase or to glycosphingolipids. It is further metabolised by ceramidase to sphingosine, which can then be phosphorylated into S1P by sphingosine kinase isoforms 1 and 2 (SphK1/2). This phosphorylation can be reverted by the S1P phosphatases SPP1 and SPP2 or irreversible degradation by S1P lyase can occur. S1P may act intracellularly or is exported out of the cell via ABC transporters or Spns2, dependent on the cell type, and may bind to one of its receptors (S1PR1–S1PR5) to initiate G-protein mediated signaling. S1P modulates key processes of vascular pathogenesis which involve but are not restricted to modulation of cell proliferation and migration and regulation of vascular tone and immune functions.

S1P is secreted, stored, and exported by the cells of the vessel wall, VSMCs, and ECs, respectively. Recent observations highlight the critical role of the putative S1P transporter spinster homolog 2 (Spns2) in endothelial S1P release and in lymphocyte trafficking [33, 34]. In other cell types, that is, breast cancer and mast cell, the ABC (ATP-binding cassette) transporter family members ABCC1 and ABCG2, known regulators of inflammatory processes, facilitate export of S1P across the cell membrane [35, 36]. S1P regulates a diverse range of cellular processes that are important in immunity, inflammation, and inflammatory disorders [37, 38]. Once secreted, most of the S1P binds to albumin or serum lipoproteins [39]. Whether this carrier-bound serum S1P or rather locally produced S1P is important for the diverse cellular functions is a matter of current debate [40]. The metabolism and distinct vascular functions of S1P are highlighted in Figure 1.

## 3. Interactions of S1P Receptors and Thrombin Receptors Affect Endothelial Function

Endothelial cells synthesize and secrete large amounts of S1P and contribute substantially to generating the high S1P level present in the blood [40, 41]. Of the five S1PRs, endothelial cells express S1PR1, S1PR2, and S1PR3 [29]. S1P modulates diverse endothelial activities including proliferation [42], survival [43], migration [44], and regulation of proinflammatory responses [45] and controls the endothelial barrier function [46–49]. S1PR1 is highly expressed in endothelial cells [50] and regulates cytoskeletal structure, migration, and vessel maturation [51, 52]. In S1PR1 receptor deficient embryos, blood vessels were incompletely covered by VSMCs, indicating that S1PR1 also regulates vascular maturation [53]. Thus, S1PR1 appears to mediate predominantly physiological functions while particularly S1PR2 regulates inflammatory endothelial responses and is upregulated during inflammatory conditions such as atherosclerosis [45, 54]. These assumptions are in agreement with recent observations of varying S1P concentrations resulting in differential receptor activation [55] and the differential regulation of S1PR1 and S1PR2 expression during conditions of hyperglycemia-induced endothelial cell dysfunction [56].

**Figure 2:**
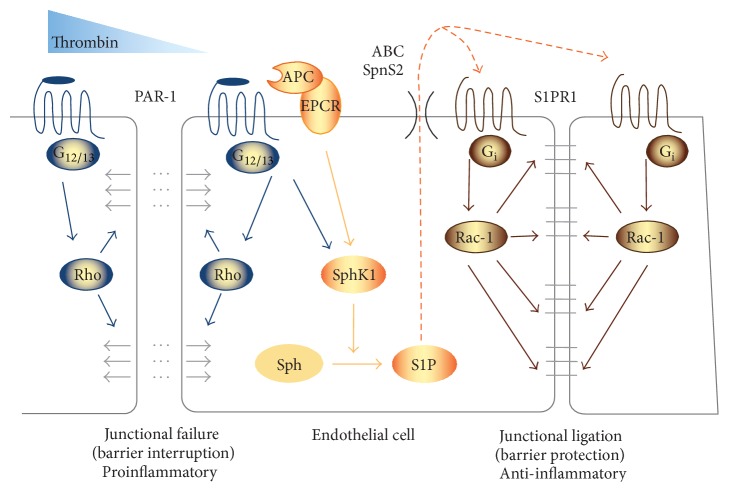
Thrombin effects in endothelial cells involve S1P signaling. Activation of the classical thrombin receptor PAR-1 interrupts endothelial barrier integrity by induction of endothelial contraction through stimulation of G_12/13_ subunit and Rho signalling pathway (left). Conversely, thrombin also induces expression of SphK1 and increases S1P generation. This involves PAR-1-induced signaling via activated protein C (APC) and the endothelial protein C receptor (EPCR). S1P in turn transactivates S1PR1 leading to G_i_-dependent activation of the Rac-1 signaling pathway. This effect improves endothelial integrity to counteract and limit thrombin-induced endothelial damage and vascular leakage (right).

A key regulator of endothelial function is the coagulation system with factors such as thrombin known to affect its permeability [57] as well as endothelial inflammation [58]. Thrombin causes induction of endothelial cell contraction and disruption of endothelial barrier integrity via the PAR-1 receptor [57]. This involves signaling through the endothelial protein C receptor and includes cross talk with the S1PR system [59]. S1P/S1PR signaling can counteract this detrimental effect of thrombin and appears to protect from vascular leakage and tissue damage such as edema formation [60]. Thus, thrombin may enhance endothelial S1P generation and signaling within the endothelium to limit its own actions of inducing vascular leakage via mutual PAR-1 mediated S1P/S1PR1 actions (Figure 2).

In certain systemic diseases such as sepsis, signaling through PAR-1 exerts multiple and partly opposing functions. This has been attributed to either promoting dendritic cell-dependent coagulation and inflammation or reducing sepsis lethality due to protein C activation and involves regulation of the balance between differential vascular S1PR (S1PR1/S1PR3) signaling pathways [59]. Thus, not only PAR signaling but also S1P actions in endothelial cells appear to involve opposing mechanisms and cellular effects. On the one hand, S1P enhances barrier integrity to counteract thrombin-mediated disturbance of permeability to restore vascular homeostasis after injury; on the other hand, it synergizes with thrombin in upregulating the expression of TF in endothelial cells [61]. Thereby, S1P may enhance generation of thrombin under proinflammatory conditions such as atherosclerosis. In this context, a recent study from Campos et al. is of interest, which showed that the functional S1P receptor antagonist fingolimod [62] reduces infarct size and enhances blood-brain barrier integrity in rodent models of stroke [63]. To determine whether this observation, besides an effect on barrier function, involves direct thrombotic or antithrombotic mechanisms of S1P signaling requires further investigations. The mechanistic studies which directly link S1P and its receptors to the thrombin or FXa receptors, their (patho)physiological actions, and associated signaling pathways are summarized in Table 1.

## 4. Role of Coagulation Factor-S1P Interactions in the Vessel Wall

Proliferation and migration of vascular VSMCs are fundamental features in physiological processes such as maturation of blood vessel [64] as well as during vascular lesion formation [65]. Numerous growth factors and inflammatory molecules like cytokines regulate VSMCs proliferation and migration. Early studies also suggested a function of S1P for DNA synthesis and migration in VSMCs [66]. Since then, S1P has rapidly been gaining attention as a key regulator of VSMCs functions and vascular development as well as a critical factor for vascular damage. Like endothelial cells, VSMCs obtained from different vascular beds express S1PR1, S1PR2, and S1PR3 receptors [67–71]. Kluk and Hla reported that S1P via activation of S1PR1 significantly stimulates both proliferative and migratory responses for VSMCs [70]. This is in agreement with the observation that S1P induces VSMCs migration through a G*α*i-linked, Ras- and PI3-K-coupled, ERK1/2-dependent process [71]. A further role of S1PR2 receptor in vascular physiology and pathology has been established through regulation of intracellular signaling pathways, such as Rho GTPase, the phosphatase PTEN, and VE-cadherin pathways [72]. Nodai et al. found high mRNA levels of the receptors S1PR2 and S1PR3 in rat VSMCs [73]. They suggest that predominantly S1PR3 stimulates expression of COX-2 through mechanisms involving calcium-dependent PKC and Src-family tyrosine kinase [73].

The relevance of S1P in the regulation of vascular permeability, lymphocyte trafficking, and vascular development is well documented* in vivo* [41]. S1PR1 deficiency resulted in impaired vascular maturation [74] whereas SphK1 and SphK2 null mice have shown disturbed angiogenesis resulting in embryonic lethality [75]. Furthermore, Kono et al. reported that S1PR1, S1PR2, and S1RP3 function coordinately during embryonic angiogenesis [76]. Taken together, these studies suggest S1P governs physiological vascular homeostasis and is also an important mediator during pathophysiological conditions such as inflammation.

The coagulation system has been well recognized as a key regulator of inflammation. An interaction between thrombin-induced PAR-1 signaling and the S1P system via enhanced expression of SphK1 and elevated S1P synthesis has first been observed in epithelial and in endothelial cells [77]. In addition, the S1P system has been suggested as a downstream component of thrombin signaling also in other cell types. Work from Niessen et al. revealed a critical role of cross talk between PAR-1 and the S1PR3 receptor in dendritic cells in the amplification of inflammation during sepsis [78]. Further studies indicate direct involvement of thrombin in regulating key processes of cellular proinflammatory responses in VSMCs. This involves activation of classical inflammatory transcription factors such as NF-*κ*B [79], but also immune regulators that have more recently become of interest, that is, the forkhead-box-O transcription factor family [80].

**Figure 3:**
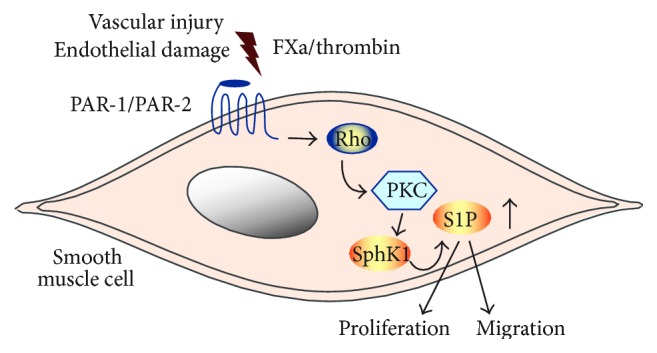
The coagulation proteases thrombin and activated factor X (FXa) enhance S1P synthesis and release from vascular smooth muscle cells (VSCMs). During vascular injury, local generation of thrombin and FXa enhances the synthesis and the release of S1P via activation of PAR-1 and PAR-2, respectively. This signaling pathway involves activation of the small GTPase Rho and PKC signaling leading to transcriptional upregulation of SphK-1. The resulting S1P activities regulate proliferation and migration of VSMCs.

In addition to thrombin, FXa can independently activate PAR-1 and PAR-2. Recent work from our laboratory has shown that FXa regulates transcription of SphK1 and elevates S1P biosynthesis in human vascular smooth muscle cells (Figure 3) [81]. This stimulatory effect, observed in cultured cells, was seen at FXa concentrations (3 to 30 nM) which have been shown to occur during thrombus formation* ex vivo* [26]. Expression of SphK1 by FXa was attenuated by inhibitors of the Rho-associated kinase and of classical PKC isoforms. In addition, FXa caused activation of the small GTPase RhoA in human smooth muscle cells. This is particularly interesting, because small GTPases are known to play key roles in mediating signaling responses of the S1P receptor [82], suggesting a mutual interaction of S1P receptor-initiated signaling and regulation of S1P synthesis. Interestingly, FX/FXa appears to be already present within human carotid artery plaques (plaque material is well known to be highly thrombotic) and colocalizes with SphK1 expression [81]. The presence of active coagulation factors in atherosclerotic tissue has also been shown by others [83]. This observation suggests a close relation between coagulation factor signaling and progression of the atherothrombotic disease. Whether possible antiproliferative or antiatherogenic actions of the novel oral coagulation inhibitors involve affecting SphK1 expression and possibly modification of S1P-mediated signaling in patients requires further investigations.

## 5. Release Mechanisms of S1P from Activated Platelets

The biological effects of S1P released from activated platelets in the vasculature include inhibition of platelet aggregation [84], angiogenesis, vascular development, and thrombosis-related vascular diseases such as the acute coronary syndrome [47, 85, 86]. Platelets were originally suggested to be the prime source of plasma S1P, because they exhibit high SphK activity. In human platelets, SphK2 is the predominant isoform [87]. Surprisingly, however, although platelets do express S1P receptors [88] during* in vitro* platelet function testing such as light transmission aggregometry, S1P does not appear to function as a potent direct platelet agonist [89].

**Figure 4:**
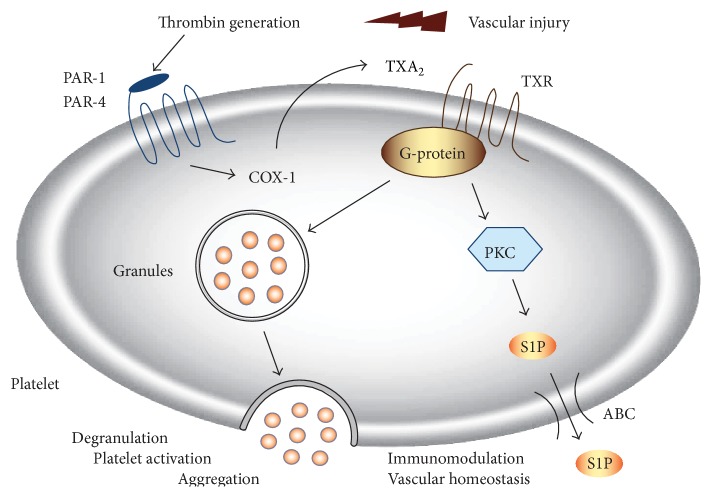
Thrombin-stimulated S1P secretion in human platelets. Thrombin triggers COX-1-mediated synthesis of thromboxane A_2_ (TXA_2_) via activation of platelet PAR-1. Consecutive TX release and activation of thromboxane A_2_ receptor (TXR) enhances platelet degranulation and aggregation. In parallel, TXA_2_-TXR signaling stimulates platelet S1P secretion. This pathway involves activation protein kinase C (PKC) and appears to be mediated by an ATP-dependent transport mechanism (ABC, ATP-binding cassette transporter). In a paracrine manner, platelet-derived S1P may modulate endothelial and immune cell responses at sites of injury.

Due to lack of S1P lyase activity in platelets [90], S1P abundantly accumulates intracellularly. However, S1P plasma levels in thrombocytopenic mice were found to remain largely unchanged [91], suggesting that resting platelets may not substantially contribute to circulating S1P concentrations in plasma. Platelets release huge amounts of S1P during blood clotting or upon direct activation with agonists of PKC signaling like thrombin [92, 93]. Work from our laboratory suggests a critical role of thromboxane in regulating the release of S1P from human platelets [89]. Secretion of S1P was induced after activation of platelets with potent agonists such as thrombin or selective PAR-activating peptides (PAR-APs) or with a high concentration of collagen. This effect was largely prevented after inhibition of thromboxane formation by classical inhibitors of cyclooxygenase-1 (COX-1), such as aspirin, diclofenac, or ibuprofen (Figure 4 and [89]). Thus, one pathway mediating release of platelet-derived S1P after platelet activation depends on COX-1-derived thromboxane.

Since S1P represents an amphiphilic anion, its translocation across the plasma membrane supposedly does require active transport proteins. As mentioned above, several studies in various cell types point to the involvement of a transporter of the ABC family [94, 95]. However, the biological functions of these proteins are by far not completely understood. In activated platelets, S1P secretion was affected by several compounds that are known to inhibit members of the multidrug resistance protein (MRP/ABCC) subfamily of ABC transporters [89]. A variety of transporters including MRPs are expressed in platelets that exert important functions for translocation and storage of signaling molecules [96–98]. Further studies are required to identify the proteins involved in the export of S1P out of the cells. The elucidation of the mechanisms of S1P release would also provide the opportunity of pharmacological modulation of the transport process.

## 6. Interactions of the S1P-PAR System for Inflammatory Monocyte Responses

During vascular inflammation, monocytes secrete several proinflammatory cytokines and adhesion molecules, a response mechanism which facilitates recruitment and adherence to the inflamed and activated endothelium [99]. Thrombin is one of the key factors controlling the migratory and secretory behavior of monocytes [100]. Human peripheral blood monocytes predominantly express PAR-1 and PAR-3 [101]. Interestingly, during differentiation into macrophages, that is, by colony-stimulating factors, the expression levels of PAR-1, PAR-2, and PAR-3 are highly elevated indicating dynamic adaptation mechanisms of the system [101]. A recent report indicates that monocytes from patients with antiphospholipid syndrome expressed PAR-1 to PAR-3 but not PAR-4 [102]. Other authors have described a role for PAR-4 in the release of inflammatory markers from monocytes, such as IL-6 [103]. Thus, different PARs may be differentially regulated in response to various stimuli during vascular pathogenesis.

S1P is a recently recognized novel regulator also of monocyte functions [104, 105]. Human monocytes express all five S1PRs at the mRNA and protein levels [106], possibly mediating the regulation of monocyte apoptosis and chemotaxis [107]. In leukocytes, S1P contributes to P-selectin-dependent rolling through endothelial S1PR3 [108]. In dendritic and endothelial cells, involvement of S1P in the signaling pathways of the prototypic thrombin receptor PAR-1 has been suggested [80]. However, little information is to date available about a possible cross talk between S1P and PARs in monocytes. Recent data from our laboratory provide evidence that (i) S1P directly enhances expression of the thrombin receptors PAR-1 and PAR-4 in human monocytes and that (ii) this results in enhanced PAR-4-mediated chemotaxis and elevated generation of COX-2 in response to thrombin [109].

**Figure 5:**
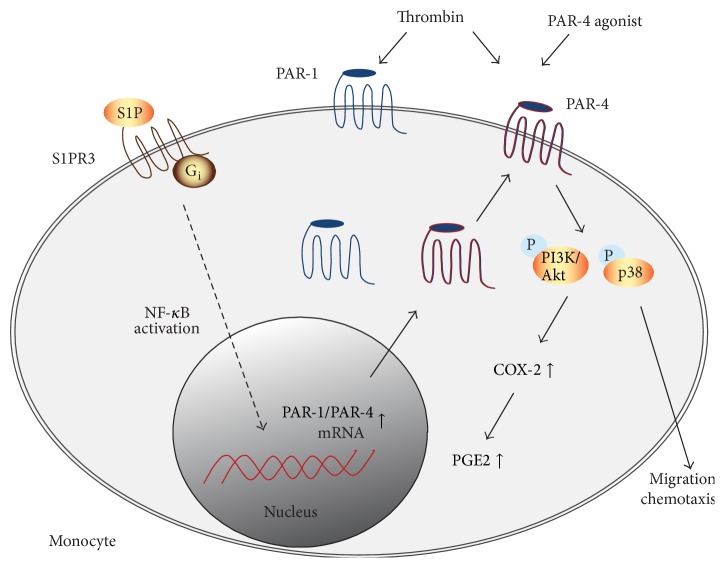
Interactions of S1PRs and PARs in monocyte function. In human monocytes, S1P significantly enhances expression of the thrombin receptors PAR-1 and PAR-4 at the mRNA and protein level. Elevation of PAR-1/PAR-4 abundance appears to be mediated via activation of S1PR3 and results in increased migration of the monocyte toward thrombin. These responses are associated with PI3K/Akt-mediated expression of COX-2. At sites of vascular injury, increased levels of S1P, for example, released from aggregating platelets, may enhance the inflammatory response of local monocytes, thereby modulating tissue-targeted events such as thrombosis and vessel injury.

S1P induced PAR-1 and PAR-4 mRNA and total protein expression in human monocytes and U937 cells in a concentration- and time-dependent manner, respectively. However, only PAR-4 cell-surface expression was increased significantly by S1P, whereas cell-surface PAR-1 remained unaffected. This response was associated with activation of the Akt, ERK1/2, and p38 pathway and induction of COX-2 but not COX-1. PAR-4-mediated induction of COX-2 was prevented by pharmacological inhibition of the PI3 kinase pathway and incubation of human monocytes with S1P resulted in an enhanced PAR-4-dependent chemotaxis response to thrombin. Thus, S1P enhances monocyte responses to thrombin via upregulation of PAR-4 protein and cell-surface expression, which promotes migration and COX-2 abundance. These studies establish a direct link between S1P receptor activation and regulation of thrombin receptor expression in human monocyte and the subsequent cellular responses to thrombin. This mechanism may facilitate monocyte recruitment to sites of vessel injury and inflammation (Figure 5).

## 7. Summary and Perspective

Taken together, complex (patho)physiological interactions between blood coagulation factors and S1P and their respective signaling receptors are being increasingly recognized (see Table 1). This involves regulation of endothelial, smooth muscle, and immune cell functions. Of particular interest for the clinic is the use of new selective modulators of the S1P-S1PR signaling system such as fingolimod as therapeutic agents. In the cardiovascular system, the role of S1P as therapeutic target or as a potential biomarker in cardiovascular diseases is still unclear. For example, the role of S1P levels and release, that is, from thrombin-activated platelets during myocardial infarction, is not finally defined to date. Recent studies indicate that S1P levels substantially vary during cardiovascular disease entities [110, 111]. An important future issue is the definition of circulating S1P levels in defined study populations as well as in clinical cohorts such as patients with acute coronary syndrome. The clinical relevance and therapeutic potential of altering S1P levels or receptor activity in atherothrombosis associated diseases is to date unclear and warrants future studies.

## Figures and Tables

**Table 1:** Mechanistic studies which directly link S1P and its receptors to the thrombin or FXa receptors PAR-1 to PAR-4, their (patho)physiological actions, and associated signaling pathways.

Receptor(s)/stimuli	G-protein binding	Signaling pathway	Physiological action(s)	Reference

S1P/thrombin	Not described	NF-*κ*B, EGR-1/ERK1/2	Enhanced tissue factor expression in endothelial cells	[59]

S1P/thrombin	GIT1 and GIT2	Focal adhesion kinase (FAK)/Src	Regulation of endothelial barrier function	[46]

S1PR3/PAR-1 (via SphK1)	G_12/13_	IL-1B	Induces tissue factor production, inflammation, and coagulation	[37]

S1PR1 and S1PR3/PAR-1	G_i_; G_12/13_	Rac-1/Rho	Inflammatory responses	[76]

S1P/PAR-1 (via SphK1)	G_12/13_	NF-*κ*B	Regulation of endothelial function	[75]

SphK1/FXa via PAR-1 and PAR-2	Gq	Rho-kinase, PKC	Mitogenesis and migration of VSMCs	[79]

S1P/FXa	G*α*; G_12/13_	Rho-A/GTPases	Proliferation/survival	[80]

S1PR3/PAR-4	G_i_	Akt, p38 MAPK	Migration, chemotaxis	[107]

## Abbreviations

ABC: Adenosine triphosphate-binding cassette transporter
APC: Activated protein C
COX-1: Cyclooxygenase-1
EGR-1: Early growth response protein 1
EPCR: Endothelial protein C receptor
FAK: Focal adhesion kinase
FXa: Activated coagulation factor X
NF-*κ*B: Nuclear factor-*κ*B
PAR-1/PAR-2/PAR-4: Protease-activated receptor 1/2/4
p38 MAPK: p38 mitogen-activated protein kinases
PI3K: Phosphatidylinositol-3-kinases
PAR-4AP: PAR-4 activating peptide
PGE2: Prostaglandin E2
PKC: Protein kinase C
Rac-1: Ras-related C3 botulinum toxin substrate 1
Rho: Ras-homologue GTPase family member
S1P: Sphingosine-1-phosphate
S1P1–S1P3: S1P receptors 1 to 3
Sph: Sphingosine
SphK-1: Sphingosine kinase-1
Spns2: Spinster homolog 2
TXA_2_: Thromboxane A_2_
W146: S1PR1 antagonist
CAY: S1P3 antagonist
LY: An inhibitor of PI3K upstream of Akt
SB: p38 MAPK inhibitor.
